# Immunology and Infection by Protozoan Parasites

**DOI:** 10.1155/2015/504951

**Published:** 2015-03-19

**Authors:** Edecio Cunha-Neto, Christophe Chevillard, Mauricio Martins Rodrigues, Marcelo T. Bozza

**Affiliations:** ^1^Heart Institute (Incor), University of São Paulo School of Medicine, 05403-000 São Paulo, SP, Brazil; ^2^INSERM and Université Aix-Marseille, Marseille, France; ^3^Escola Paulista de Medicina, Universidade Federal de São Paulo, 04044-010 São Paulo, SP, Brazil; ^4^Departamento de Imunologia, Instituto de Microbiologia, Universidade Federal do Rio de Janeiro, 21941-902 Rio de Janeiro, RJ, Brazil

Protozoan infection is the cause of diseases of high morbidity and mortality. Most are non-self-limiting chronic infections and neglected diseases; emergent antimicrobial-resistant strains pose a substantial problem; for many of them treatment either is highly toxic or has limited effectiveness. Vaccine development is still a formidable task and there is no licensed vaccine for human protozoan infection. The key to the control of protozoan infection is the understanding of the host immune response to protozoan parasites, which will guide the development of effective vaccines and immunotherapeutic agents. In this special issue, there are important studies on the immunology of* T. cruzi* infection, tegumentary and visceral leishmaniasis, malaria, and toxoplasmosis, both in patients and in animal models, which promote the understanding of immunopathological/immunoprotective parameters in these diseases.

The reviews by J. M. Álvarez and colleagues and by E. Cunha-Neto and C. Chevillard cover fundamental findings, mechanisms, and questions concerning the immune response and pathology of mouse and human disease. In this line, L. G. Nogueira and colleagues characterize the myocardial expression of transcriptional factors involved in effector CD4+ T cell differentiation and the characteristic cytokines produced by the distinct lymphocyte subsets and demonstrate a profound predominance of local Th1 response. L. C. J. Abel and coworkers characterized the proinflammatory effects of glicophosphatidyl inositol-anchored* T. cruzi* mucin (GPI-mucins) on human immune cells. It was observed that IL-12 production by GPI-mucins was dependent on IFN-*γ* and CD40-CD40L interactions. F. C. Dias and colleagues investigated the HLA-G expression in tissues and HLA-G 147 3′ UTR polymorphic site typing in patients presenting Chagas disease and the role of the mouse functional homolog in* T. cruzi* infection. F. Vorraro and colleagues studied the susceptibility to* T. cruzi* of mouse strains previously selected based on inflammatory and antibody responses to complex antigens. Q. Miao and M. Ndao review the potential impact of* T. cruzi* infection on the status of host lipid metabolism. The role of lipid mediators in* T. cruzi* infection is explored in two research articles. A. M. C. Canavaci and colleagues revisit the pathological role of 5-lipoxygenase in the acute infection of mice and A. D. Malvezi and coworkers investigate the role of cyclooxygenase and lipoxins in macrophage invasion by* T. cruzi*. In mouse models of chronic myocardial disease by* T. cruzi*, I. R. Pereira and colleagues show that treatment with infliximab, an anti-human TNF monoclonal antibody, reduced the frequency of mice with arrhythmias and cardiac fibrosis, while M. B. de Cuba and coworkers show the beneficial effect of cholinergic stimulation in reducing inflammation and fibrosis in the heart. Importantly, A. F. Araújo and coworkers demonstrate the beneficial effects of prophylactic vaccination using* T. cruzi* genes against acute and chronic pathologies caused by myotropic strains of the parasite. W. H. K. Cabrera and colleagues have characterized mouse lines selected for maximal (AIRmax) or minimal (AIRmin) acute inflammatory reaction and for high (HIII) or low (LIII) antibody (Ab) responses to complex antigens. Resistance to* T. cruzi* infection was found in female and in high responder lines AIRmax and HIII. It was correlated with enhanced production of IFN-*γ* and nitric oxide production by peritoneal and lymph node cells, in HIII males and females. Moreover, an Ab production QTL marker mapping to mouse chromosome 1 significantly cosegregated with survival after acute* T. cruzi* infection.

In the* Leishmania* field, M. F. Lopes and colleagues here review the interface between* Leishmania* and phagocytes, cells that are both targets and effector of the anti-*Leishmania* immune response. The study by E. Svensjö and colleagues suggests that a serine peptidase (ISP-2) from* L. major* regulates macrophage phagocytosis by inhibiting the pericellular release of proinflammatory kinins from surface bound kininogens. C. M. V. Vendrame and coworkers have shown that insulin-like growth factor- (IGF-) I decreased nitric oxide production but increased arginase expression and activity, which lead to increased* Leishmania* parasitism. However, IGF-I did not result in altered cytokine levels. Moreover, stimulation with IGF-I induced phosphatidylserine exposure on amastigotes led to increased arginase activity in macrophages, and this process was not blocked by anti-TGF-*β* antibodies. M. D. T. Carvalho and colleagues investigated risk markers of* L. infantum* visceral leishmaniasis and found that lipoprotein and triglyceride levels are risk factors for development of visceral leishmaniasis. They also found that genetic polymorphisms at the Lpl and Ppar*γ* were associated with differential levels of lipoproteins and thus related to the disease outcome. I. Naouar and coworkers investigated granzyme-positive CD4+ T cells activated by* L. major* antigens, in* Leishmania*-healed subjects. They found that even CD4+CD25+CD127^dim/−^ Tregs expressed granzyme after exposure to* Leishmania* antigens.

A number of manuscripts published in this issue provide important new knowledge in the important field of malaria. A review from H. Zheng and colleagues highlighted important aspects of preerythrocytic stages biology and the immune evasion strategies of malaria parasites. The unraveling of these escape mechanisms may aid the development of a vaccine against malaria. In this same direction, the manuscript by the group of J. Huang and colleagues generated a new mouse model to study the immunity to these preerythrocytic stages. This new model employed transgenic MHC-I Kd mice. The results of their studies indicate that protective antimalaria immunity induced by radiation-attenuated sporozoites of* Plasmodium yoelii* in MHC-I-Kd-Tg mice is mediated by CS protein-specific, Kd-restricted CD8+ T cells. E. S. Fernandes and colleagues studied the effect of TRPV1 antagonism by capsazepine during mouse infection with* Plasmodium berghei* ANKA. Their result indicated that there was a modulation of the innate immune response in mice infected but it did not affect parasitemia. Two other studies dealt with malaria pathogenesis. L. S. Ortolan and coworkers described new predictive criteria to study the pathogenesis of malaria-2 associated ali/ards in mice. This method for accurately identifying mice suffering from ALI/ARDS before death will allow the use of this model to study the pathogenesis of this disease. Finally, J. C. Sánchez-Arcila and colleagues evaluated whether intestinal parasites coinfection would alter plasma cytokines profile elicited in acute malaria in subjects from endemic area of Brazil. They concluded that the infection with intestinal parasites (mainly protozoan) does not significantly modify the pattern of cytokine production in individuals infected with* P. falciparum* and* P. vivax*.

Finally, A. S. Machado and colleagues investigated the immunopathogenesis of toxoplasmosis in parturients and nonpregnant women. They measured the synthesis of Th1 and Th2 cytokines by mononuclear cells after culture with live* T. gondii* and identified Th17 (CD4+) and Tc17 (CD8+) cells in toxoplasma-seronegative and toxoplasma-seropositive parturient and nonpregnant women. They observed a lower level of IL-17-expressing CD4+ and CD8+ T lymphocytes in cultures of cells from seronegative and seropositive parturient and nonpregnant women that were stimulated with tachyzoites. It has been shown that the cytokine pattern and IL-17-expressing CD4+ and CD8+ T lymphocytes may have important roles in the inflammatory response to* T. gondii*, thus contributing to the maintenance of pregnancy and control of parasite invasion and replication. A. S. Machado and coworkers studied immunological and hematological biomarkers of ocular congenital toxoplasmosis in infants, finding differential immune parameter networks in each clinical group (active and cicatricial ocular toxoplasmosis).

*Edecio Cunha-Neto* *Edecio Cunha-Neto* *Christophe Chevillard* *Christophe Chevillard* *Mauricio Martins Rodrigues* *Mauricio Martins Rodrigues* *Marcelo T. Bozza* *Marcelo T. Bozza*

